# Safety of Raloxifene in Hemodialysis Patients

**DOI:** 10.5812/ijem.6285

**Published:** 2012-09-30

**Authors:** Didy Jacobsen

**Affiliations:** 1 Geriatric Department, Radboud University Medical Center, Nijmegen, The Netherlands

**Keywords:** Raloxifene, Hemodialysis

Dear Editor,

With interest I read the article from Saito and colleagues about “the effects of raloxifene on bone metabolism in hemodialysis patients with type 2 diabetes” (1). The article addresses the possibility of treatment with raloxifene for osteoporosis in these patients. Raloxifene, a selective estrogen receptor modulator could be a good option, because it has no known renal side effects like bisphosphonates. Additionally, bisphosphonate use in chronic kidney disease is associated with adynamic bone disease, compared with a low bone turnover (2). In another trial in a group of 17 postmenopausal hemodialysis patients raloxifene improved lumbar spine Bone Mineral Density (BMD) by 2.6% in 53% of the patients while 70% of the control group consisting of 10 aged-matched women showed a reduction in BMD by 4%. The effects of raloxifene on serum calcium and serum iPTH level suggest it improves bone resorption (3). Primary, in these patients treatment of secondary hyperparathyroidism should be performed as is recommended (4). However of concern is the safety of raloxifene treatment in this patient group with multimorbidity. Long term studies should be performed to determine the efficacy and safety of raloxifene in hemodialysis patients.

Diabetes mellitus and chronic kidney disease both have a negative effect on bone metabolism and there is an increased risk of vascular complications. In the dialysis population the incidence of myocardial infarction and stroke is 5- to 15 fold higher (5) and cardiovascular mortality is 10- to 30-fold higher (6) than seen in the general population (7-9).

In this hemodialysis population with or without diabetes mellitus I would expect an increased risk of fatal stroke with raloxifene therapy. Taking into account the data of the RUTH-trial in 10101 postmenopausal women with a mean age of 67 years, with a high Framingham Stroke Risk Score (FSRS) ( ≥ 13), a 75% increased risk of raloxifene–associated fatal stroke was found. (HR 1.75; 95% CI, 1.01-3.02) (10). The FSRS includes the classical risk factors for cardiovascular disease including age, sex, systolic blood pressure, total and high density lipoprotein cholesterol, diabetes mellitus, smoking and echocardiogram based left ventricular hypertrophy (11-13). This score was originally validated for people aged up to 75 years (14). The FSRS also seems to underestimate the vascular risk in hemodialysis patients. The prevalence of traditional cardiovascular disease risk factors is very high in this hemodialysisgroup, but seems not to explain all of the increased cardiovascular risk (15).

I would recommend a longer term randomized, double-blind, placebo-controlled trial in hemodialysis patients (subgroup analysis with or without diabetes mellitus). Raloxifene versus placebo should be given for 3 years with attention to the change in BMD and monitoring fractures and vascular complications. First it should be shown that the number needed to treat to prevent fracture is clearly smaller than the number needed to harm in a clinical trial of the duration of the required raloxifene treatment before raloxifene can be safely recommended as common practice in the treatment of osteoporosis in hemodialysis patients. Saito et al. performed an important preliminary study that warrants the investment of such a trial to reach the quality standard of safe and evidence based therapy in this vulnerable patient group.
